# Noninvasive NMR/MRS Metabolic Parameters to Evaluate Metabolic Syndrome in Rats

**DOI:** 10.3390/diagnostics12071621

**Published:** 2022-07-04

**Authors:** Khin Thandar Htun, Krit Jaikumkao, Jie Pan, Aye Thidar Moe Moe, Nuttawadee Intachai, Sasivimon Promsan, Anusorn Lungkaphin, Monruedee Tapanya, Duanghathai Pasanta, Montree Tungjai, Siriprapa Kaewjaeng, Hong Joo Kim, Jakrapong Kaewkhao, Christopher Lai, Suchart Kothan

**Affiliations:** 1 Center of Radiation Research and Medical Imaging, Department of Radiologic Technology, Faculty of Associated Medical Sciences, Chiang Mai University, Chiang Mai 50200, Thailand; khinthandar_htun@cmu.ac.th (K.T.H.); krit.ja@cmu.ac.th (K.J.); ayethidar_moemoe@cmu.ac.th (A.T.M.M.); nuttawadee.i@cmu.ac.th (N.I.); monruedee.t@cmu.ac.th (M.T.); duanghathai.pas@cmu.ac.th (D.P.); montree.t@cmu.ac.th (M.T.); siriprapa.k@cmu.ac.th (S.K.); 2 Shandong Provincial Key Laboratory of Animal Resistant Biology, College of Life Sciences, Shandong Normal University, Jinan 250014, China; 3 Department of Physiology, Faculty of Medicine, Chiang Mai University, Chiang Mai 50200, Thailand; sasivimon_promsan@cmu.ac.th (S.P.); anusorn.lungka@cmu.ac.th (A.L.); 4 Department of Physics, Kyungpook National University, Daegu 41566, Korea; hongjoo@knu.ac.kr; 5 Center of Excellence in Glass Technology and Materials Science (CEGM), Faculty of Science and Technology, Nakhon Pathom Rajabhat University, Nakhon Pathom 73000, Thailand; jakrapong@webmail.npru.ac.th; 6 Health and Social Science Cluster, Singapore Institute of Technology, 10 Dover Drive, Singapore 138683, Singapore; chris.lai@singaporetech.edu.sg

**Keywords:** MRI, ^1^H MRS, ^1^H NMR, HFD, metabolic syndrome

## Abstract

(1) Background: Ectopic fat deposition and its effects, metabolic syndrome, have been significantly correlated to lifestyle and caloric consumption. There is no specific noninvasive evaluation tool being used in order to establish clinical markers for tracing the metabolic pathway implicated in obesity-related abnormalities that occur in the body as a result of a high-fat diet (HFD). The purpose of this work is to investigate in vivo ectopic fat distribution and in vitro metabolite profiles given by HFDs, as well as how they are inter-related, in order to find surrogate metabolic biomarkers in the development of metabolic syndrome utilizing noninvasive approaches. (2) Methods: Male Wistar rats were divided into a standard normal chow diet, ND group, and HFD group. After 16 weeks of different diet administration, blood samples were collected for proton nuclear magnetic resonance (^1^H NMR) and biochemical analysis. Magnetic resonance imaging/proton magnetic resonance spectroscopy (MRI/^1^H MRS) was performed on the abdomen, liver, and psoas muscle of the rats. (3) Results: Visceral fat showed the strongest relationship with blood cholesterol. Although liver fat content (LFC) was not associated with any biophysical profiles, it had the highest correlation with metabolites such as (-CH_2_)_n_ very-low-density lipoprotein/low-density lipoprotein (VLDL/LDL), lactate, and N-acetyl glycoprotein of serum ^1^H NMR. HFD showed no obvious influence on muscle fat accumulation. Acetoacetate, N-acetyl glycoprotein, lactate, (-CH_2_)_n_ VLDL/LDL, and valine were the five possible metabolic biomarkers used to differentiate HFD from ND in the present study. (4) Conclusions: Our study has validated the influence of long-term HFD-induced ectopic fat on body metabolism as well as the metabolic profile deterioration both in vivo and in vitro.

## 1. Introduction

Obesity is one of the most serious public health concerns of the twenty-first century. Since the 1980s, the prevalence of global obesity has been high, reaching triple digits in many countries that make up the European Region of the World Health Organization (WHO). Obesity, along with the health hazards that come with it, has become a public health crisis in both industrialized and developing countries [1,2]. Notification of high levels of triglycerides (TGs) in very-low-density lipoprotein/low-density lipoprotein (VLDL/LDL) is a main foreteller for cardiovascular disease (CVD) risk assessment [3]. Excess or improper fat deposition in organs other than adipose tissue has long been thought to play a role in the development of metabolic diseases such as insulin resistance and type 2 diabetes. According to new cross-sectional interventional evidence, dietary fat composition may play a crucial role in ectopic fat deposition, which may increase the likelihood of metabolic syndrome disease [4]. The connection between regional fat distribution and metabolic changes generated by a high-fat diet (HFD) has become a contentious issue.

It has been established that the macronutrient composition of a diet linked to ectopic fat can disrupt metabolic processes and can also affect organ function [5]. As a reason, it may be a valuable attribute for distinguishing between persons who have a healthy metabolism and those who have a disordered metabolism. Fatty acid that transfers from adipose tissue to nonadipose tissues is a major contributor to metabolic changes [6,7]. Understanding how the HFD influences ectopic fat distribution, and the metabolite changes that result, could help in a better understanding of the metabolic mechanism that produces metabolic syndrome. Therefore, to determine whether a subject has excess fat distribution that comes from an excessively HFD and its related abnormal metabolite conditions, further diagnostic assessments need to be established.

Indeed, the interaction between highly metabolically active visceral fat and an accelerated rate of de novo lipogenesis in hepatic steatosis may enhance the loss of hepatic insulin sensitivity and is the major cause of hyperglycemia [8,9]. In order to determine the effect of greater morbidity and mortality caused by pathogenic visceral fat and fatty liver in obesity, a noninvasive and reliable evaluation of visceral fat and hepatocellular lipid content is necessary. A number of methods for assessing body composition have been developed, but some of them, such as body dissection and chemical analyses, are invasive measurement procedures [10,11]. For identifying metabolic profiles, precise and noninvasive diagnostic approaches have recently become crucial. There is no unique noninvasive monitoring technique for identifying the metabolic process involved in obesity-related disorders that emerge in the body as a consequence of an HFD. Having exceptional soft tissue resolution and inherently high contrast between fat and water makes magnetic resonance imaging (MRI) an ideal technique for assessing adipose tissues and muscle [12]. This study provides the data needed to locate the metabolic pathway implicated in metabolic alterations brought on by ectopic fat in the body as a result of an HFD.

Metabolomics research based on bioanalytical chemistry has been shown to be an effective method for detecting inter- and intravariability in metabolic pathways altered by various digestions [13]. Proton nuclear magnetic resonance (^1^H NMR) spectroscopy is the most widely used metabolomics technology, especially in cancer, diabetes, inborn errors of metabolism, and cardiovascular disease research [14]. The combination of multivariate data analysis with nontargeted ^1^H NMR spectroscopy profiling of the various biofluids has to be a novel approach for metabolomic detection in obesity because of its high and simultaneous detection of a wide range of chemicals [15,16].

The etiology of human obesity-related metabolic anomalies is remarkably similar in both obese and lean rats, as a point of reference [17]. Although both Wistar and Sprague Dawley rats can be utilized as models for HF diet-induced obesity, Wistar rats show stronger and earlier metabolic effects and are good models for dissecting aspects of physiological control that are hard to analyze in humans [18,19]. 

We intended to see how metabolomics and ectopic fat content changed in noninvasive ways in moderately different diet administrations (low-fat diet and HFD) in order to develop diagnostic markers, particularly for use as obesity-related disease assessment tools. Not only are blood biochemical parameters used, but body fat composition and metabolomics biomarkers are also characterized as alternative determinant biomarkers in noninvasive obesity clinical management. We discovered promising in vitro metabolite biomarkers in blood and ectopic fat depots in various anatomies in this work, along with correct relations between certain biochemical profiles.

## 2. Materials and Methods

### 2.1. Animal Experiment

A total of 26 male Wistar rats (180–200 g) aged 2 months were obtained from the National Animal Center, Salaya Campus, Mahidol University, Thailand. They were randomly divided into two dietary groups, normal chow diet, ND (n = 13) and HFD (n = 13). All experimental rats were housed under controlled temperature (25 ± 1 °C) and lighting in a 12:12 hour light/dark cycle (lights on 06:30–18:30) with food and water ad libitum. After 7 days of acclimatization, the rats in the ND group were provided standard rat chow (C.P. Mice Feed Food No. 082;) with 4.02 kcal/g energy rate and the HFD group consumed 5.35 kcal/g energy rate containing 57.60 fat % of total energy percentage [20]. The compositions of the ND and HFD are shown in Table 1 and Table 2.

### 2.2. Ethical Considerations

The animal facilities and protocols were approved by the Laboratory Animal Care and Use Committees at the Faculty of Medicine, Chiang Mai University, Chiang Mai, Thailand and followed the National Institute of Health guidelines on the ethical use of animals (Permit number: 07/2562). 

### 2.3. Biophysical Characteristics

All animals were allowed free access to water and fresh food every day. Body weight and food intake were recorded daily, which was then converted into total daily energy intake.

### 2.4. Biochemical Blood Analysis

After 16 weeks of different food administration, blood samples from both groups were obtained by cutting the tail tip after overnight fasting. All serum samples were stored at −80 °C for further analysis. All laboratory examinations were carried out by using a fully automated analyzer (Architect ci8200, Abbott Diagnostic, Lake County, IL, USA). Blood lipid cholesterol, TGs, and fasting glucose (FG) were measured. 

### 2.5. Magnetic Resonance Imaging for Abdominal Fat Detection

After 16 weeks of different food administration, the rats were anesthetized with 40 mg/kg pentobarbital (single dose) via intraperitoneal injection. The abdomens of both groups were scanned in prone position by a 1.5 Tesla, Ingenia, Philip MR machine (Philips Healthcare, Amsterdam, The Netherlands) under anesthetic conditions. The 3 mm thickness T1-weighted axial images were collected with the following parameters: Repetition time (TR) = 450 ms, echo time (TE) = 8 ms, field of view (FOV) = 200 mm. Afterward, an axial slice was taken at the level that was free from the liver or buttock adipose tissue and was saved in digital imaging and communications in medicine (DICOM) format for fat quantification.

The image was analyzed using the Medical Image Processing, Analysis, and Visualization (MIPAV, National Institutes of Health, Bethesda, MA, USA) software package equipped with a semiautomatic segmentation technique which converts grayscale pixels into binary images of black and white, based on the signal intensity-based histogram-thresholding method [21]. The software uses the grayscale range (0–255). Mathematical morphology helps to determine the tissues according to different grayscales being used together with a histogram (intensity versus pixel count) for intensity threshold determination. The area with high signal intensity, or that which appears brighter, represents abdominal adipose tissue which was set as the threshold to exclude the nonrelevant organs and tissue in the image [22].

**Figure 1:**
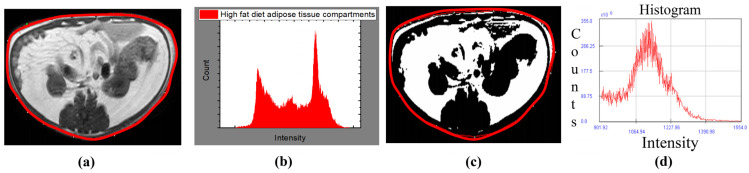
T1 weighted image analysis by MIPAV software; (**a**) transverse abdominal image with ROI redlining, (**b**) grayscale-related histogram of T1-weighted MR image with intensity on the *x*-axis and pixel count on the *y*-axis, (**c**) segmental transverse abdominal fat image with ROI redlining, and (**d**) histogram of total abdominal tissue with intensity on the *x*-axis and pixel count on the *y*-axis.

The pixel value that appeared as white in segmental images represents abdominal adipose tissue content, while black pixels represent mesenteric air and soft tissue such as muscle, blood vessels, and bony structures, as shown in Figure 1c. The visceral and subcutaneous regions were determined through the manual drawing of a region of interest (ROI) along the abdominal wall that demarcates the intraperitoneal and extraperitoneal regions [23]. Afterward, the abdominal fat percentage (Abd fat %) and visceral fat percentage (Vis fat %) were measured first. Next, the subcutaneous fat percentage (SC fat %) was calculated by subtracting these values.

### 2.6. Single-Voxel Proton Magnetic Resonance Spectroscopy (^1^H MRS) for Liver and Psoas Muscular Fat Detection

**Figure 2:**
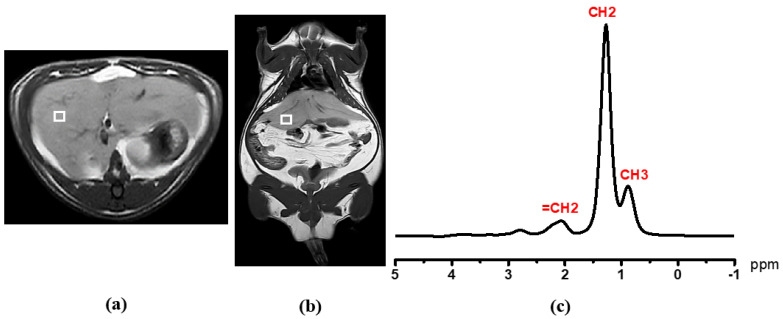
MR spectra acquisition: Liver image with single voxel (5 × 5 × 5 mm^3^) on (**a**) axial image and (**b**) coronal image. In graph (**c**), MRS spectrum obtained from single-voxel PRESS sequence. Methyl (-CH_3_) peak at 0.9 ppm and methylene (-CH_2_) peak at 1.3 ppm. White square: voxel.

**Figure 3:**
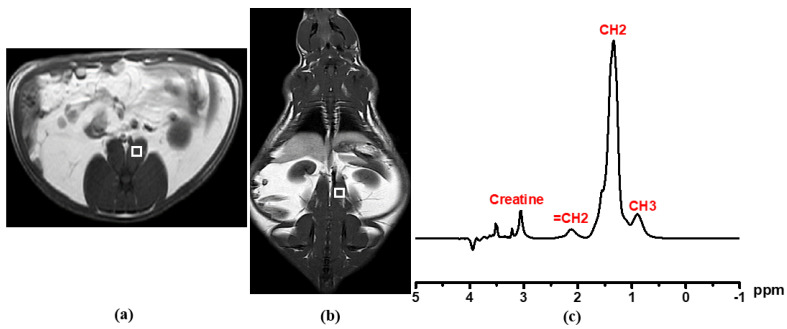
MR spectra acquisition: Psoas muscle image with single voxel (5 × 5 × 5 mm^3^) on (**a**) axial image and (**b**) coronal image. In graph (**c**), MRS spectrum obtained from single-voxel PRESS sequence. Methyl (-CH_3_) peak at 0.9 ppm, methylene (-CH_2_) peak at 1.3 ppm, unsaturated methylene (=CH_2_) at 2.1 ppm, and creatine at 3.04 ppm. White square: voxel.

Before ^1^H MRS acquisition, 3 mm thickness T1 and T2 MR images of three planes (axial, sagittal, and coronal) were scanned by TFE and TSE sequences for the exact voxel localization using a 1.5 T (Ingenia, Philips Medical Systems, Amsterdam, The Netherlands) equipped with a head coil. Liver metabolite spectrum without water suppression was taken by a single-voxel point resolved spectroscopy (PRESS) technique with 5 × 5 × 5 mm^3^ voxel size on the right lobe of liver (Couinaud lobe segment V–VIII) that avoided the bile ducts and large vascular structures, as shown in Figure 2. The spectrum was acquired with repetition time (TR) = 2000 ms, echo time (TE) = 31 ms, number of signal averaging = 128. For muscular lipid metabolite detection, the voxel was carefully positioned on the left psoas muscle as shown in Figure 3. 

Metabolite signals with and without water suppression were analyzed for metabolite identification and quantification. Liver fat content (LFC) and psoas muscular fat content were processed by Tarquin 4.3.10 free software. The CSV format spectrum fitting was carried out by quickly fitting the acquisition data into Tarquin software. The peak area is represented as the concentration of the center peak metabolite at a specific number of parts per million (ppm). After setting of spectrum baseline mode and treatment in an OriginPro analyzer, auto-peak determination was carried out using a local maximum peak filtering method by a threshold height percentage setting. The resultant peaks were adjusted manually, if needed. The integration was carried out from the baseline *x*-axis. Spectrum fitting and quantification were carried out for the water peak (4.7 ppm), and the major lipid peaks -CH_3_ (0.9 ppm), -CH_2_ (1.3 ppm), and allylic (2.1 ppm) were carefully integrated with prior knowledge [24]. The T2 relaxation correction was performed using a linear least square equation to determine T2 of water and fat. The hepatic fat and muscular fat contents were obtained from area peak integration at a specific chemical shift by OriginPro 2015 (64 bits) Beta3 b9.2.196 software.

### 2.7. Proton Nuclear Magnetic Resonance (^1^H NMR) Acquisition and Analysis

Serum metabolites of ND and HFD groups were detected by the ^1^H NMR method. After centrifuging, 200 μL of supernatant fresh serum sample was dissolved by adding 400 μL of dimethyl sulfoxide-D6 (99.8% deuteration) and was mixed gently. The homogeneous solution was transferred into a 5 mm diameter high-quality NMR tube. Data were acquired using a Bruker AVANCE 500 MHz (Bruker, Bremen, Germany) with a Carr–Purcell–Meiboom–Gill (CPMG) water-suppression presaturation pulse sequence at 27 °C. A 90° pulse with number of signal averaging (NSA) of 16 was applied. A spectrum in the 0–6 ppm range was analyzed by TopSpin 4.0.7 software.

**Figure 4:**
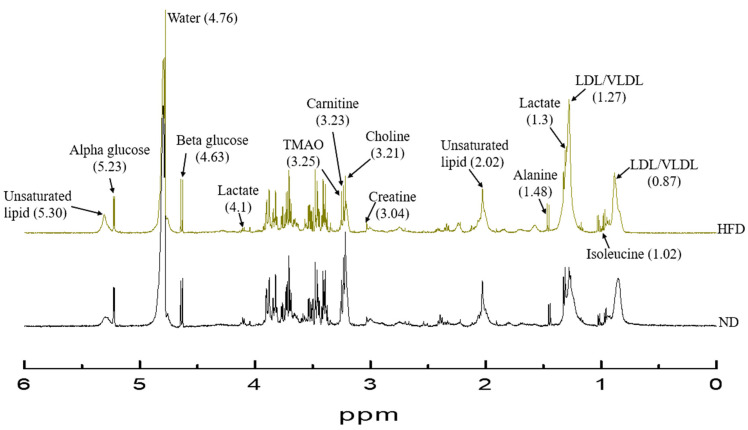
^1^H NMR spectra of normal chow diet (ND) and high-fat diet (HFD) groups.

After transforming of frequency domain spectrum pattern from the time domain free induction decay (FID) signal, the baseline and phase were corrected by using automatic code insertion. The solvent peak ppm or known metabolite peak ppm was set as a reference for calibration. The individual metabolite integrations were carried out after manual peak picking. Metabolites were identified and compared to existing literature [25]. The resulting specific peak integration seen in Figure 4 was normalized by dividing the total integration value.

### 2.8. Statistical Analysis

The Kolmogorov–Smirnov test was used to identify the degree of deviation from a normal distribution within the data set. In univariate analysis, two-sample *t*-statistics were performed to define the differences between ND and HFD groups based on biophysical profiles, biochemical results, fat contents estimated by MRI/^1^H MRS, and serum metabolomics detected by ^1^H NMR. They were presented as mean ± SD values. Pearson’s correlation analysis was carried out to verify the relationship of fat content and the model from biophysical and biochemical profiles, thus *p* < 0.05 denotes statistical significance. Multivariate statistics were carried out with principal component analysis (PCA) to look for clustering and possible confounders in the data set. Later, partial least squares discriminant analysis (PLS-DA) was used in order to make a model to discriminate between ND and HFD groups. The total amount of variation between the groups was explained by the model with R2Y (cum %) and that within the group was denoted as R2X (cum %). The predictive ability of the model was determined by cross-validation and denoted as Q2 (cum %). The variable with a significant difference in univariate analysis (two-sample *t*-test) and a PLS-DA variable importance in projection (VIP) value exceeding 1 was defined as the strongest variable that contributes to the group discrimination [26,27]. Statistical analysis was performed using IBM SPSS version 17 and OriginPro 2015.

## 3. Results

The results of the two groups were determined 16 weeks after the consumption of different diets (ND and HFD).

### 3.1. Biophysical Parameters

Although daily HFD food intake (in weight) was almost 4% less than that of ND, the total daily energy intake by HFD rats was 27.8% greater than that of ND rats, accounting for 118.8 ± 16.14 kcal, *p* < 0.05 (Table 3). Body weight increased in both groups. At the end of 16 weeks of providing different food, the HFD group reached 733.33 ± 132.46 g which was approximately 35.80% (193.30 g) higher than that of the ND group with *p* < 0.05.

### 3.2. Blood Biochemical Measurements

According to biochemical assay results, only the total cholesterol level in HFD (125.41 ± 14.48 mg/dL) was noticeably different from ND rats (92 ± 20.14 mg/dL) with *p* < 0.05. The blood TG and FG levels did not significantly change between the two groups. Both biophysical and biochemical characteristics are shown in Table 3.

### 3.3. Abdominal Fat Compartments and Laboratory Characteristics

The total Abd fat % obviously increased in HFD with 43.60 ± 7.31%, but in ND had only increased 31.31 ± 12.65%. Additionally, HFD showed about 40.41 ± 7.80% visceral fat compartments which was about 36.98% higher than ND visceral fat content. There was no significant variation in subcutaneous adipose tissue between the groups (Table 4).

**Figure 5:**
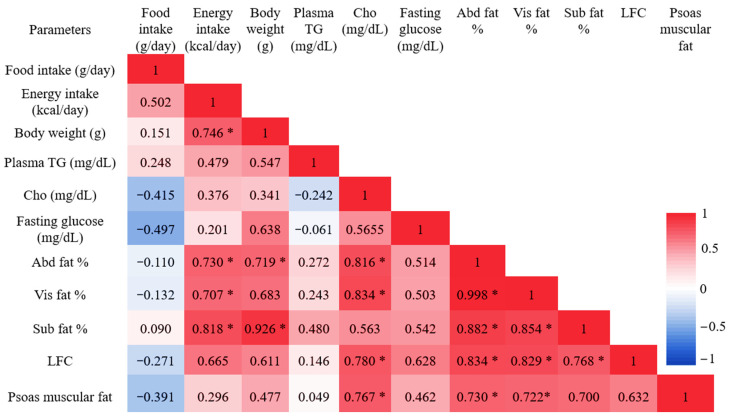
All data are presented as correlation coefficient “r” values with the color scales of Pearson linear correlation between in vivo, biophysical, and biochemical data. Significant values are shown as * *p* < 0.05. TG, triglyceride; Cho, cholesterol; FG, fasting glucose; Abd fat %, abdominal fat percentage; Vis fat %, visceral fat percentage; SC fat %, subcutaneous fat percentage; and LFC, liver fat content.

Pearson’s correlation in Figure 5 shows that all fat percentages in the abdominal region, Abd fat %, Vis fat %, and SC fat %, were strongly and significantly associated with daily energy intake with r = 0.730, r = 0.707, and r = 0.818, *p* < 0.05 respectively. Vis fat %, Abd fat %, and SC fat % correlated strongly with body weight with r = 0.719, r = 0.683, *p* < 0.05 and r = 0.926, *p* < 0.001. In biochemical association, Vis fat % had the strongest relationship with blood cholesterol (r = 0.834, *p* < 0.05). Furthermore, abdominal fat % also was related to cholesterol as r = 0.816, *p* < 0.05. SC fat % had no association with blood biochemical data at all.

### 3.4. Liver Fat Content (LFC) and Laboratory Characteristics

In this study, both saturated and unsaturated methylene lipids in the liver, -CH_2_ (1.3 ppm) and =CH_2_ (2.1ppm), were significantly different between the two groups. The total liver fat content percentage in the HFD group was approximately 19.2-fold higher than that in the ND group (Table 4). Liver fat content was not associated with biophysical profiles (food intake, energy intake, and body weight) at all. However, it was strongly and positively associated with blood biochemical cholesterol (r = 0.780, *p* < 0.05). Individual fat content percentage in abdominal region, Abd fat %, Vis fat %, and SC fat %, had a strong relationship with liver fat according to r = 0.834, 0.829, 0.768, *p* < 0.05, respectively (Figure 5). 

### 3.5. Psoas Muscular Fat Content and Laboratory Characteristics

The total fat composition in psoas muscles of HFD seemed greater than in the ND group, but the difference was not significant. It was strongly associated with blood cholesterol (r = 0.767, *p* < 0.05), but had no relationship with any biophysical profiles. According to Pearson’s interrelation of ectopic fat accumulation in different regions, psoas muscular fat amounts strongly and directly correlated with both abdominal fat and visceral fat contents with r = 0.730, 0.722 and *p* < 0.05. However, there was no relation to subcutaneous fat amount and to liver fat content. The descriptive characteristics of different anatomic fat contents between ND and HFD groups and Pearson’s correlation results are presented in Table 4 and Figure 5.

### 3.6. ^1^H NMR Metabolomic Characteristics

A total of 24 regions (metabolic profiles) were recognized from serum ^1^H NMR spectrum analysis that was related to different diet interventions. Altogether, seven variables differed significantly based on descriptive analysis between ND and HFD groups with *p* < 0.05 occurring in all. Those metabolite variables were lactate (4.1 ppm and 1.3 ppm), acetoacetate (2.22 ppm), N-acetyl glycoprotein NAC1 (2.14 ppm), methylene lipid of VLDL/LDL (1.27 ppm), and valine (1.05 ppm and 0.996 ppm) after normalization. These trends and all metabolite comparisons are shown in Table 5. Among the significant differences between ND and HFD individuals, univariate analysis revealed that lactate (1.3 ppm) and (-CH_2_)n VLDL/LDL (1.27 ppm) had the highest increments with their VIP > 1. However, acetoacetate was shown to have the greatest downtrend.

**Figure 6:**
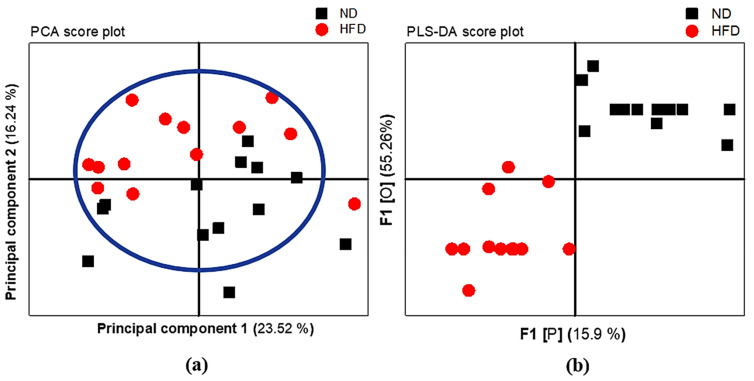
^1^H NMR variables of normal chow diet group (ND) shown in black and high-fat diet group (HFD) in red are plotted according to different body weights based on the 24 serum variables. (**a**) PCA score plot shows principal component 1 on the *x*-axis and principal component 2 on the *y*-axis, and (**b**) PLS-DA score plot shows the first predictive factor 1, F1 [P], on the *x*-axis, and the first orthogonal component 1, F1 [O], on the *y*-axis.

The PCA score plot was based on the 24 serum ^1^H NMR variables of ND and HFD rats according to different diet statuses. Each sample was represented by its metabolic profile and visualized as a single symbol of which the location was determined by the contributions of the 24 variables in the ^1^H NMR spectrum. There was a color clustering as shown in Figure 6a. This means that this model allows HFD individuals to be clearly differentiated from their ND counterparts. This model also explained the 23.52% of variation according to principal component 1 (PC1), being the largest variance within the data set, and the 16.24% of variation according to principal component 2 (PC2), being the second largest variance within the data. Multivariate PLS-DA was used to train a classification model (classifier) in discriminating between the HFD and ND individuals based on data input from their metabolic profile. The model explained that the total amount of variation within the groups was 29.93% (R2X(cum)) and, between the groups, it was 71.03% (R2Y (cum)). The predictive ability of the model was good enough to discriminate between the groups on the basis of the metabolic profile with a Q2(cum) of 75.88%. The PLS-DA score plot shows the first predictive component F1 [P] (15.9%), explaining the variation between the groups, versus the first orthogonal component F1 [O] (55.26%) that explains the variation within the groups, as seen in Figure 6b.

**Figure 7:**
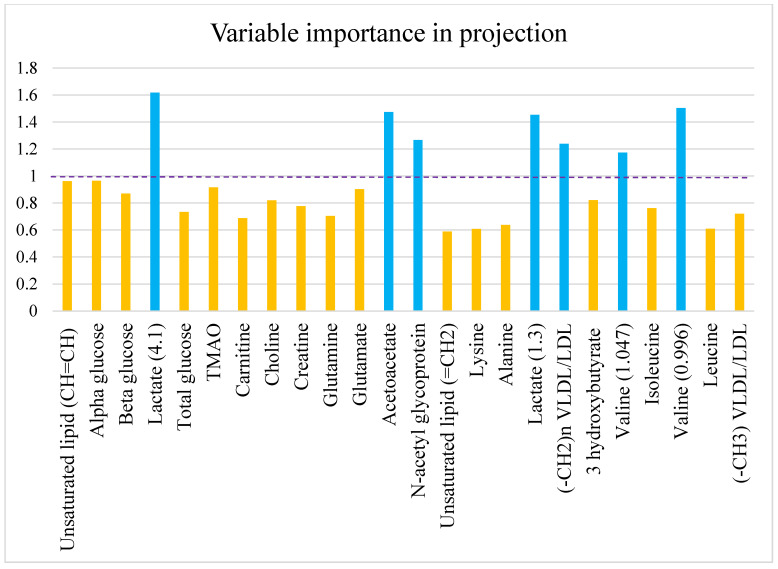
Bar chart showing the variable importance in projection (VIP) scores. Blue bars represent metabolites which have VIP > 1 and have significant differences between HFD and ND groups with *p* < 0.05. VLDL, very-low-density lipoprotein; LDL, low-density lipoprotein; TMAO, trimethylamine N-oxide.

The variables that strongly contribute to discrimination between HFD and ND individuals are determined according to significant univariate changes with *p* < 0.05 and PLS-DA VIP value > 1. In this study, N-acetyl glycoprotein (2.14 ppm), lactate (1.3 ppm and 4.1 ppm), and (-CH_2_)n VLDL/LDL (1.27 ppm) are significantly higher. However, acetate (2.22 ppm) and valine (1.047 ppm and 0.996 ppm) are lower in HFD compared to ND with VIP > 1. The seven possible metabolites that discriminate HFD from ND are shown in Figure 7. The most significant change in serum concentration between HFD and ND is observed in variable 18, representing the saturated lipid (-CH_2_)n VLDL/LDL, being about 76.02%, as shown in Table 5.

**Figure 8:**
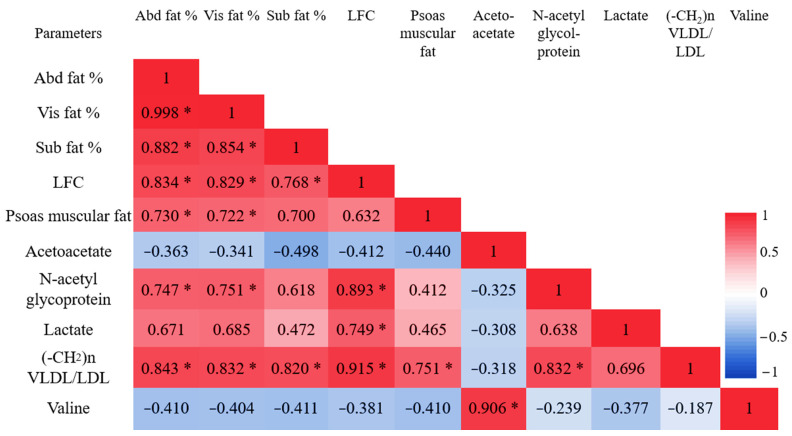
All data are presented as correlation coefficient “r” values of Pearson linear correlation between in vivo ectopic fat contents and vitro ^1^H NMR serum metabolite profiles. Significant values are shown as * *p* < 0.05. VLDL, very-low-density lipoprotein; LDL, low-density lipoprotein; Abd fat %, abdominal fat percentage; Vis fat %, visceral fat percentage; SC fat %, subcutaneous fat percentage; and LFC, liver fat content.

Being associated with NMR metabolites, all three fat depots in the abdomen, the intra-abdominal, and subdermal regions were directly proportional to both lactate (r = 0.788, 0.781, 0.739 and *p* < 0.05 in all) and saturated -CH_2_ (1.27 ppm) of lipid TG (r = 0.843, 0.832, 0.820 and *p* < 0.05), as shown in Figure 8. LFC totally correlated with methylene protons of -CH_2_ (1.27 ppm) in the ^1^H NMR spectrum results with very the strongest coefficient values being r = 0.915. Lactate and N-acetyl glycoprotein were also associated with LFC as shown in Figure 8. The muscular fat deposit was strongly related to only saturated -CH_2_ (1.27 ppm) of lipid TG (r = 0.751 and *p* < 0.05) among the other serum metabolites in this ^1^H NMR study.

## 4. Discussion

A greater knowledge of the processes that regulate adipose tissue expansion in obesity is essential for the development of future therapeutic options for obesity-related metabolic problems. This study showed that long-term HFD consumption caused higher fat accumulation in different organs, accompanied by systematic metabolic changes in multiple biochemical measures such as blood chemical profiles. The metabolic effects of the HFD were found to be due to a range of metabolic pathways, including glycolysis/gluconeogenesis, fatty acid, amino acid, ketone body, and choline metabolism issues, according to our findings. These significant metabolic variations were detected after 16 weeks of HFD ingestion.

Many studies show that the pathological effects of excessive adiposity are dependent not only on the quantity of fat, but also on the distribution of the fat mass in the body [28]. Different types of regional excessive fat distribution can be a disease concern, and abnormal body fat depots in particular parts of the anatomy may have unique metabolic features. The visceral fat, white adipose tissue surrounding the major abdominal organs, is thought to be the principal adipose depot involved in the etiology of obesity-related disorders, while the subcutaneous fat plays a less prominent role [29]. 

Despite the fact that food consumption rates varied less in this study, the HFD’s significant rise in energy intake and body weight was almost certainly due to the diet’s energy states. An et al. also found that no matter what the amount/weight of food consumed, the energy intake in HFD-fed rats was literally greater than that of ND-fed control [30]. The biophysiological metabolic mechanism deterioration may be a potential consequence of the unhealthy type of energy obtained from food ingestion. Our study has shown that the main difference in diet composition more significantly impacts lipid metabolism, as clearly seen in the higher blood cholesterol levels of the HFD group compared to higher carbohydrate ingestion in the ND group. 

Moreover, the 16-week HFD intake induced a significant increase in rat body weight that was in turn related to the obvious accumulation of fat in abdominal subcutaneous regions detected by MRI. This is possibly because subcutaneous adipose tissue has a higher preadipocyte cell population and a faster preadipocyte proliferating rate [31]. There was no statistically significant link between visceral fat content and body weight; this may be due to the smaller size of visceral adipocytes [32]. Furthermore, the quantity of adipocytes determines weight [33]. Indeed, smaller adipocytes and their smaller population in visceral fat, unrelated to BMI or gender, may indirectly noninvasively indicate their lack of significance in this study’s body weight growth.

Our noninvasive MRI findings indicate that calorie intake is the primary cause of ectopic lipid accumulation in all abdominal compartments. We made sure that the relative weight of epididymal white adipose tissue was higher in mice fed the HFD compared to the mice fed the high-carbohydrate diet [34]. In this study, the body weight and HFD energy intake are the main influences on the subcutaneous fat accumulation. Furthermore, neither the subcutaneous fat relationship to plasma TG nor the association with cholesterol and FG demonstrated the less metabolic nature of subcutaneous fat. This finding totally coincided with the weak relationship of subcutaneous fat to lipid metabolites (TG and high-density lipoprotein cholesterol: HDL-C) and showed no association with FG in young adult obesity in vivo [35]. This fact supports the fact that highly mobilized TG in subcutaneous fat, which serves as a buffer during high dietary lipid intake and protects against lipotoxicity, seems to play a role in glycemic control and therefore reduces cardiovascular risk in humans [36,37].

The enormous amount of fat packed inside the peritoneal cavity between internal organs and the torso, whereby the abdomen protrudes excessively, is probably due to intra-abdominal obesity. Our study evaluated the strongest relationship between visceral fat and blood cholesterol (r = 0.834, *p* < 0.05) among the other ectopic fat relations. This finding is consistent with our in vivo study on young adult populations in which visceral fat had the most significant and strongest relationship to blood TG and HDL-C levels when compared to abdominal and subcutaneous lipids [35]. Meek et al. also found that intra-abdominal adipocytes are less responsive to insulin’s anti-lipolytic actions, and that visceral adipose tissue-derived free fatty acid (FFA) may have more significance in vivo, reaching up to 40%. This can affect hepatic function in obesity [38]. In addition, an in vitro human study showed that omental adipocytes have high metabolic responses to adrenergic stimulation, with fewer anti-lipolytic alpha-2 adrenergic receptors (ARs) and higher lipolytic beta-AR levels [39]. Our result revealed that fat depots inside the peritoneal cavity have higher metabolic activity that mainly impacts fat metabolism compared to fat deposition in any other areas. There was a similar conclusion in studies carried out on humans. This finding supports the idea that high visceral fat, especially in young obese people, could be a dyslipidemia-treated biomarker [35]. The health risks vary depending on the compartmental obesity, even in the same degree of obesity, and particularly in intra-abdominal obesity. As a result, metabolic syndromes with diabetes and atherosclerosis illnesses may become more prevalent [36,40].

Excessive visceral fat accumulation leads to the secretion of free fatty acids into the portal vein and the secretion of pro-inflammatory adipocytokines which leads to abnormal accumulation of lipids in hepatocytes, a characteristic feature of hepatic steatosis [41,42]. Due to overloading of visceral adiposity, shown by an inability to allow subcutaneous adipose tissue metabolic compensation, the surplus energy in the subcutaneous adipose tissue is stored as an accumulation of fat at undesired sites [43]. Our results showed that 16-week HFD intake caused a significant increase in rat body weights and abdominal fat content with the concurrent accumulation of lipid droplets in the liver. Although there was no significant relation between liver fat and any biophysical profiles (body weights, energy intake) of rats, there was a strong association with blood cholesterol found in this study. This result is totally consistent with the proposed portal hypothesis, whereby the influx of excess FFA in the portal vein is attributed to the high TG accumulation in the hepatocytes and induces de novo lipogenesis that promotes the secretion of atherogenic lipoprotein cholesterol [44]. This fact was supported in our observation of HFD-induced elevations of blood cholesterol together with the TG accumulation in the liver. Our previous ^1^H MRS study also showed that the amounts of intrahepatocellular lipids in obese and overweight people were substantially greater than in normal-weight adults. In fact, they were around 9.4 and 4.1 times higher than normal-weight people, respectively. Furthermore, higher liver fat in young obese individuals may be a significant predictor of glucose metabolism and hyperglycemia [35].

Interestingly, the standard deviation observed in liver fat content value was considerably larger in the HFD group than in controls, suggesting that HFD effects on the biochemistry of rats were heterogeneous. Such heterogeneity was also observable for two rats in terms of their cholesterol levels, even though their body weight-to-cholesterol relationships were obviously inverted. This result elucidates the detailed mechanisms underlying the emergence of such heterogeneity and suggests that obese rats use excessive metabolic regulation to generate intrahepatocellular lipids via the reverse cholesterol transport system. It probably came from different feeding schemes [45,46]. However, in previous studies, the obesity-prone and obesity-resistant subsets of rats responded differently to the same HFD [47].

The HFD group had no significant increase in psoas muscle fat in this investigation. In addition, muscle fat had no association with either calorie intake or body weight. This result was slightly different than the previous human findings that higher muscular fat content was found in higher BMI young adults [35]. It appears that the overconsumption of fat has a lower likelihood of influencing muscle fat accumulation, not just in reaction to physical parameters, but also in conjunction with muscular fiber type composition and oxidative activity [48]. This may be caused by the predominance of glycolytic fiber type II composition, in which high ATPase activity with dynamic activity leads to a reduction in myocellular TG depot size, although there may be some HFD intake [49]. Our observations showed that there was no muscle fat linked with liver fat, but instead it was linked with visceral fat. This fact was associated with an increase in total body fat and visceral adiposity, which were accompanied by more intramyocellular and extramyocellular lipids (IMCLs and EMCLs) stored in obese adolescents [50]. Fonvig et al. also discovered that muscle fat deposition was highly linked to visceral fat, but was unrelated to liver function [51]. Furthermore, the impact of muscle fat on biochemical blood cholesterol changes during HFD induction was revealed in our research. The findings imply that IMCL and EMCL content in ^1^H MRS may be a risk factor for arterial stiffness in human trials [52].

In comparison to ND supply, our research found that an HFD causes some noticeable metabolite alterations. Even though biochemical data showed that glucose tributaries were not considerably higher in the HFD group, this current investigation found a huge rising trend in lactate. According to our prior young adult human investigation, higher lactate levels appear to be a possible discriminating biomarker for nonhyperlipidemic and hyperlipidemic obesity [53]. In fact, the HFD induced white adipose tissue expansion (hypertrophic adipocytes) due to fat storage within the adipocytes [54]. Instead of generating acetyl CoA through aerobic metabolism, those low-mitochondrial-function hypertrophic adipocytes in white adipose tissue activate the anaerobic glycolysis pathway [55]. Furthermore, the reduced vascularity of larger adipocytes promotes local adipose tissue hypoxia, and greater lactate production indicates the cells’ lack of adequate oxygen supply [56]. 

A minimal change in glucose and a noticeable increase in N-acetyl glycoproteins in the HFD group occurring in this study may indicate a fatty liver response to pro-inflammatory cytokines as increased production of acute phase-1 acid glycoproteins [57]. This is similar to NMR research carried out on Ningxiang type obese pigs [58]. In addition, greater glucose consumption and higher N-acetyl were discovered in our in vitro human investigation and may be a result of hyperactivation of the hexosamine pathway, especially in hyperlipidemic settings [53].

Serum lipid levels (saturated and unsaturated fatty acid chains of TG) are higher in the HFD than in the ND group, according to our ^1^H NMR investigation. The most significant metabolite with the biggest gain seen is lipid -CH_2_ protons of saturated fatty acid chains, and in particular that of VLDL and LDL. This result is consistent with biochemical analysis obtained from venous blood that shows a significant increase in cholesterol in the HFD group. Meanwhile, in human serum NMR investigations, the -CH_2_ protons of both unsaturated and saturated fatty acid chains produced high signal intensity in obese groups. Furthermore, those fatty acid chains exhibited a substantial linear association between TC, TG, and LDL laboratory values [53]. Increased serum VLDL/LDL levels imply an overproduction by the liver and increased exportation of TG containing VLDL particles into the bloodstream, which are subsequently converted to LDL. This finding supports the HFD-induced lipid metabolic pathway’s imbalanced hepatic de novo lipogenesis and fatty acid oxidation rates. Toledo et al. reported that fatty liver altered lipoprotein composition with or without diabetes [59]. Our findings are consistent with a positive correlation between hepatic fat fraction and pro-atherogenic lipidemia, as seen by increased large VLDL and small dense LDL concentrations in obese adolescents with normal glucose tolerance [60]. Furthermore, our PLS-DA analysis revealed that -CH_2_ lipids and lactate were potentially upregulated biomarkers having a VIP score of greater than 1. This is reinforced by the fact that those two metabolites have a positive link with ectopic lipid content, which is especially prevalent in the abdominal and hepatic areas.

Acetoacetate, another metabolite with a VIP score > 1, dramatically downtrended in HFD when compared to ND. The enzyme hydroxymethylglutaryl CoA (HMG-CoA) lyase produces this first ketone body mostly in the liver mitochondria during overspill of acetyl CoA from fatty acid oxidation and certain amino acids [61]. In instances of insulin resistance, fasting, or high-fat eating (nutritional ketosis), an increase in serum ketone levels indicates that tissue is utilizing fat for energy rather than glucose [62]. Our acetoacetate results contradict this fact. This finding is consistent with obesity, as significant increases in total carnitine and lysine levels in blood metabolomics, along with carnitine palmitoyltransferase 1A (CPT1A) deficiency, are linked to a slow beta oxidation rate and hypoketotic hypoglycemic conditions [63,64]. No apparent differences in serum glucose show that glucose use through the glycolysis pathway is normal in both groups. Hepatic fat oxidation can be measured using ketogenesis as a surrogate. In obese persons with fatty livers, the rate of ketogenesis is reduced by an increase in hepatic TG loading, and a decreased turnover rate is shown with severe insulin resistance, presumably contributing to hypoketonemia, when compared to lean controls in animal models [65,66]. The fat-derived ketones, such as acetoacetate, are produced from inefficient hepatic fat oxidation and are utilized for lipid generation [67]. However, more research is needed to clarify the physiological implications of hepatic futile cycling in vivo.

## 5. Conclusions

To summarize, more high-energy fatty dietary ingestion led to an increase in overall body weight. Our noninvasive MRI in vivo analysis of local ectopic fat depots in the abdominal, intra-abdominal, and subcutaneous areas shows that the energy level of a high-fat meal is closely related to all three local ectopic lipids (Abd fat %, Vis fat %, and SC fat %). Only cholesterol level in blood is correlated to Abd fat % and Vis fat %, and there is no relation with SC fat %. It has been proven that higher TG buffering in subcutaneous fat lowers lipotoxic effects. None of the systemically functioning fat depots, whether intrahepatic or intramuscular, is reliant on food or energy, although cholesterol metabolism is most likely to be. Regardless of adiposity, the high-fat meal has a lower impact on FG, but considerably promotes lipid metabolism, which corresponds to the cardiovascular risk biomarker. Furthermore, the substantial connection results between visceral fat and intrahepatic TG levels in our investigation supported the portal theory of body physiological energy adjustment. The untargeted serum metabolomics in vitro model distinguished the effect of the HFD from the normal condition. Our metabolomics study determined that lactate and lipid -CH_2_ (VLDL/LDL) are positive trend biomarkers, and that acetoacetate will be the downstream dominant metabolic profile used to distinguish the HFD-induced obesity from normal ones. These metabolic determinants can be used as biomarkers to track metabolism during metabolic changes. This provides the foundation for correcting obesity mechanisms and aids in the creation of novel anti-obesity drugs. The consequence of long-term HFD-induced ectopic fat on body metabolism and metabolic profile impairment was validated in our in vivo and in vitro research.

## Figures and Tables

**Table 1:** Composition of normal chow diet.

Composition		Normal Chow Diet	
	g (Gram)	kcal (Kilocalorie)	%E (Energy Percentage)
Carbohydrate	495.30	1981.20	51.99
Fat	83.70	753.30	19.77
Protein	269.00	1076.00	28.24
Vitamins	65.40	-	-
Fiber	34.30	-	-
Total	947.70	3810.50	100
kcal/g		4.02 kcal/g	

**Table 2:** Composition of high fat diet.

Composition		High Fat Diet	
	g (Gram)	kcal (Kilocalorie)	%E (Energy Percentage)
Carbohydrate	190.76	763.04	14.27
Fat	342.24	3080.16	57.60
Protein	353.60	1414.40	26.45
Cholesterol	10	90	1.68
Vitamins	85.19	-	-
DL-Methionine	3	-	-
Fiber	13.21	-	-
Yeast powder	1	-	-
Sodium chloride	1	-	-
Total	1000	5347.60	100
kcal/g		5.35 kcal/g	

**Table 3:** Descriptive characteristics of biophysical and biochemical profiles between normal chow diet, ND, and high-fat diet, HFD, groups.

Parameters	ND	HFD	*p*-Value
	Mean ± SD	Mean ± SD	
Food intake (g)	23.1 ± 0.97	22.2 ± 3.02	0.50
Energy intake (kcal/day)	92.96 ± 3.89	118.8 ± 16.14	<0.05 *
Body weight (g)	540 ± 42.43	733.33 ± 132.46	<0.05 *
Blood TG (mg/dL)	95.61 ± 22.08	104.05 ± 30.65	0.60
Cholesterol (mg/dL)	92 ± 20.14	125.41 ± 14.48	<0.05 *
FG (mg/dL)	113.09 ± 18.56	124.12 ± 23.51	0.39

All data are presented as mean and standard deviation (SD) values. Significant values are shown as * *p* < 0.05. ND, normal chow diet; HFD, high-fat diet; TG, triglyceride; and FG, fasting glucose.

**Table 4:** Descriptive characteristics of different fat contents between normal chow diet, ND, and high-fat diet, HFD, groups.

Parameters	ND	HFD	*p*-Value
	Mean ± SD	Mean ± SD	
Abd fat %	31.31 ± 12.65	43.60 ± 7.31	<0.05 *
Vis fat %	29.50 ± 11.42	40.41 ± 7.80	<0.05 *
SC fat %	1.81 ± 1.78	3.19 ± 2.39	0.211
LFC	3.14 ± 1.39	60.40 ± 12.90	<0.001 **
Psoas muscular fat	3.35 ± 3.01	6.25 ± 0.72	0.111

All data are presented as mean and (SD) standard deviation (SD) values. Significant values are shown as * *p* < 0.05, ** *p* < 0.00. Abd fat %, abdominal fat percentage; Vis fat %, visceral fat percentage; SC fat %, subcutaneous fat percentage; and LFC, liver fat content. ND, normal chow diet; and HFD, high-fat diet.

**Table 5:** Serum variables resolved by ^1^H NMR of ND and HFD groups.

No.	Assigned Metabolites	ppm, δ	ND	HFD	*p*-Value	Change %
			Mean ± SD	Mean ± SD		
1	Unsaturated lipid (CH=CH)	5.3	0.01 ± 0.003	0.011 ± 0.004	0.591	9.64%
2	Alpha glucose	5.23	0.019 ± 0.004	0.02 ± 0.002	0.113	10.05%
3	Beta glucose	4.63	0.022 ± 0.004	0.024 ± 0.002	0.193	7.94%
4	Lactate	4.1	0.047 ± 0.01	0.065 ± 0.011	<0.05	34.96%
5	Total glucose	3.35–3.92	0.4 ± 0.035	0.416 ± 0.033	0.261	3.83%
6	TMAO	3.25	0.016 ± 0.003	0.018 ± 0.002	0.125	9.82%
7	Carnitine	3.23	0.022 ± 0.003	0.022 ± 0.002	0.717	1.71%
8	Choline	3.21	0.037 ± 0.006	0.033 ± 0.005	0.097	−10.05%
9	Creatine	3.04	0.006 ± 0.001	0.008 ± 0.004	0.299	18.67%
10	Glutamine	2.45	0.013 ± 0.005	0.013 ± 0.003	0.7	−4.30%
11	Glutamate	2.34	0.012 ± 0.004	0.01 ± 0.003	0.285	−13.18%
12	Acetoacetate	2.22	0.006 ± 0.003	0.003 ± 0.001	<0.05	−50.94%
13	N-acetyl glycoprotein	2.14	0.029 ± 0.004	0.036 ± 0.007	<0.05	22.49%
14	Unsaturated lipid (=CH_2_)	2.02	0.046 ± 0.010	0.048 ± 0.011	0.696	3.63%
15	Lysine	1.91	0.011 ± 0.005	0.012 ± 0.003	0.686	5.80%
16	Alanine	1.48	0.016 ± 0.004	0.016 ± 0.002	0.774	−2.16%
17	Lactate	1.3	0.089 ± 0.017	0.121 ± 0.049	<0.05	36.13%
18	(-CH_2_)n VLDL/LDL	1.27	0.048 ± 0.025	0.085 ± 0.03	<0.05	76.02%
19	3 hydroxybutyrate	1.17	0.012 ± 0.004	0.011 ± 0.004	0.596	−7.05%
20	Valine	1.047	0.005 ± 0.001	0.004 ± 0.001	<0.05	−18.03%
21	Isoleucine	1.02	0.004 ± 0.001	0.003 ± 0.001	0.264	−11.41%
22	Valine	0.996	0.01 ± 0.002	0.007 ± 0.001	<0.05	−25.79%
23	Leucine	0.96	0.014 ± 0.002	0.014 ± 0.007	0.881	2.16%
24	(-CH_3_) VLDL/LDL	0.87	0.041 ± 0.019	0.043 ± 0.01	0.765	4.65%

Calculated using two independent-sample *t*-tests and presented as mean and standard deviation (SD). Significant values are shown as *p*-value. Percentage change is the increase (+) or decrease (−) in the mean in the HFD group with respect to the ND group. ND, normal chow diet; HFD, high-fat diet; VLDL, very-low-density lipoprotein; LDL, low-density lipoprotein; and TMAO, trimethylamine N-oxide.
